# Long-term depressive symptom trajectories and related baseline characteristics in primary care patients: Analysis of the PsicAP clinical trial

**DOI:** 10.1192/j.eurpsy.2024.27

**Published:** 2024-03-27

**Authors:** Maider Prieto-Vila, César González-Blanch, Francisco J. Estupiñá Puig, Joshua E.J. Buckman, Rob Saunders, Roger Muñoz-Navarro, Juan A. Moriana, Paloma Rodríguez-Ruiz, Sara Barrio-Martínez, María Carpallo-González, Antonio Cano-Vindel

**Affiliations:** 1 Department of Experimental Psychology, Cognitive Processes and Logopedics, Faculty of Psychology, Complutense University of Madrid, Madrid, Spain; 2 Mental Health Centre, University Hospital “Marqués de Valdecilla” – IDIVAL, Santander, Spain; 3 Department of Personality, Assessment and Clinical Psychology, Faculty of Psychology, Complutense University of Madrid, Madrid, Spain; 4 Research Department of Clinical, Centre for Outcomes and Research Effectiveness, Educational and Health Psychology, UCL, London, UK; 5 iCope – Camden and Islington Psychological Therapies Services, Camden & Islington NHS Foundation Trust, London, UK; 6 Department of Personality, Assessment and Psychological Treatments, Faculty of Psychology, University of Valencia, Valencia, Spain; 7 Department of Psychology, University of Cordoba, Cordoba, Spain; 8 Health Service of Madrid, Madrid, Spain

**Keywords:** depressive symptom, growth mixture modeling, longitudinal analysis, primary care, trajectories

## Abstract

**Background:**

There is heterogeneity in the long-term trajectories of depressive symptoms among patients. To date, there has been little effort to inform the long-term trajectory of symptom change and the factors associated with different trajectories. Such knowledge is key to treatment decision-making in primary care, where depression is a common reason for consultation. We aimed to identify distinct long-term trajectories of depressive symptoms and explore pre-treatment characteristics associated with them.

**Methods:**

A total of 483 patients from the PsicAP clinical trial were included. Growth mixture modeling was used to identify long-term distinct trajectories of depressive symptoms, and multinomial logistic regression models to explore associations between pre-treatment characteristics and trajectories.

**Results:**

Four trajectories were identified that best explained the observed response patterns: “recovery” (64.18%), “late recovery” (10.15%), “relapse” (13.67%), and “chronicity” (12%). There was a higher likelihood of following the recovery trajectory for patients who had received psychological treatment in addition to the treatment as usual. Chronicity was associated with higher depressive severity, comorbidity (generalized anxiety, panic, and somatic symptoms), taking antidepressants, higher emotional suppression, lower levels on life quality, and being older. Relapse was associated with higher depressive severity, somatic symptoms, and having basic education, and late recovery was associated with higher depressive severity, generalized anxiety symptoms, greater disability, and rumination.

**Conclusions:**

There were different trajectories of depressive course and related prognostic factors among the patients. However, further research is needed before these findings can significantly influence care decisions.

## Introduction

Depression is a common reason for consultation in primary care centers around the world [1]. Despite there being many clinically recommended treatment options [2], most individuals remain untreated or are not able to access evidence-based treatments [3]. Therefore, integrating such treatments into primary care has become a major international healthcare priority [4]. Initiatives in England [5], Canada [6], Australia [7], Norway [8], and Spain [9] to integrate psychological therapies into primary care have demonstrated that they are effective, cost-effective, and able to be utilized at scale [10].

Despite the efficacy of these treatments, a considerable proportion of patients do not improve or achieve a sustained recovery [11–14]. Even when patients do achieve recovery, a large number experience relapse or recurrence of depression [15, 16]. These phenomena are not new; it has been argued for many decades that large proportions of patients with depression will not get better with treatment and large proportions will either experience chronic difficulties or relapse in the years after treatment [17]. However, few studies have investigated the heterogeneity of the course of depression both during therapy and beyond, and this might hold promise for improving long-term outcomes. Modeling the trajectories across time could identify subgroups of patients at risk of poorer outcomes that might then be offered an alternative means of managing their depression, delivering more personalized care [18].

Prior studies that have modeled the trajectory of symptom change during treatment in primary care for people with depression or anxiety disorders have used routinely data collected from the IAPT services in the United Kingdom [12, 13]. These studies found four distinct trajectories/subgroups of change in depressive symptoms: three of them characterized by improvement (small or large) at different moments of the treatment and one characterized by no response or chronicity. However, neither of these studies was able to investigate trajectories during follow-up in the months or years after therapy. A review of observational studies conducted in primary care investigated the proportions of patients in four predetermined subgroups based on their outcomes: recovery, late recovery, relapse, and chronicity [19]. They found that the proportions of patients in each subgroup varied across the studies, with between 35 and 60% of participants experiencing some sort of recovery, between 10 and 17% having a chronic course, and between 7 and 65% experiencing a relapse. However, this review did not include studies that modeled the trajectories of change. Population studies with large samples and longitudinal follow-up have modeled the trajectories of depressive symptoms, identifying four common trajectories: constantly high or low depressive symptoms and trajectories characterized by increasing or decreasing depressive symptoms over time. Notably, the most prevalent trajectory is constantly low depressive symptoms, observed in more than 70% of the population [20–22].

One of the benefits of using methods to identify individuals following distinct trajectories of change is that the association between pre-treatment patient characteristics and the trajectory classes can be investigated. There are a number of important indicators associated with prognosis which can be easily measured in primary care before treatment for depression has commenced, foremost among them is the overall severity of depression [11–13, 23]. Other related factors are also associated with prognosis following treatment, including comorbid anxiety symptoms, panic, or somatization symptoms [12, 13, 23]. Other factors appear to be evidenced as prognostic factors, such as employment status [24, 25] and marital status [15, 26], and there is a group of factors that are not typically evaluated in many studies, but which have been found to be associated with worse prognosis: higher disability [13, 19]; higher suicidal risk or behavior [15, 25], higher rumination, cognitive, and attentional biases [27, 28]; and higher anhedonia [29].

Despite the relevance of this area of research, many of the prior studies have been conducted with a narrow group of the population with depression treated in primary care, having largely been conducted in just two countries (i.e., United Kingdom or Netherlands) based on the routine practice datasets of IAPT services or in a randomized controlled trial (RCT) with a small sample size in which a limited set of variables were measured, usually focusing only on clinical variables and disability. This may limit the generalizability to other populations or settings. Therefore, the aims of the current study were as follows: (1) to identify distinct trajectories of long-term changes (12 month follow-up) in depression symptoms in a large sample of patients from an RCT delivered in Spanish primary care settings and (2) to explore the association between baseline patient characteristics and specific trajectories following changes in depression symptoms during the 1-year follow-up.

## Method

### Participants

The data used for the current study were collected as part of the PsicAP clinical trial [9], in which patients were randomly allocated to receive either treatment as usual (TAU) and Transdiagnostic – cognitive behavioral treatment (TD-CBT) or TAU alone. In total, 1061 patients with emotional disorders (anxiety, depression, or somatization) were recruited across 22 primary care centers of the Spanish National Health System. TAU was delivered by the patient’s general practitioner (GP) and consisted of psychopharmacotherapy (antidepressants [ADM] or anxiolytics) and/or informal counseling. The TD-CBT was delivered by a clinical psychologist and involved seven group sessions [30]. Patients included in the trial were aged from 18 to 65 years, scored above the cut-off points on one or more of the screening scales for depression, anxiety, or somatoform disorder (PHQ-9 ≥ 10; GAD-7 ≥ 10; PHQ-15 ≥ 5, respectively) and were excluded if they reported: severe symptoms of depression (PHQ-9 ≥ 24); high level of disability (SDS ≥ 26); recent suicidal behavior; were already receiving psychological treatment; had difficulties understanding Spanish; had a diagnosis of substance dependence disorder, or a severe psychological disorder (i.e., personality disorders, eating disorders, bipolar disorder, or a psychotic condition) confirmed by an interview developed by a clinical psychologist. The study was conducted in accordance with the principles of the Declaration of Helsinki and following the Spanish Law on Data Protection (EUDRACT: 2013–001, 955–11). The study protocol was approved by the National Ethics Committee and the Spanish Agency of Medicines and Medical Devices (code: ISRCTN58437086) [31].

For the purpose of the present study, the eligibility criteria for inclusion were that patients had to demonstrate clinically significant depression symptoms (PHQ-9 ≥ 5) at baseline assessment, and complete the pre-treatment, post-treatment, plus at least one follow-up (3, 6, 12 months) assessment to provide sufficient data for the modeling approach. There were no found statistical differences between the analytical sample for the current study and all participants who started the trial, except on age and marital status (see Supplementary Table 1). A flowchart of the sample is detailed in Supplementary Figure 1.

### Measures

The variables used in the analyses are described in Table 1.

**Table 1.** Employed questionnaires information

Variable	Questionnaire	Information on measurement
Depression	Patient Health Questionnaire depressive module (PHQ–9) [32, 33]	The scale is based on DSM–IV criteria for major depressive disorder, containing nine items on a Likert scale from 0 (*not at all*) to 3 (*nearly every day*), with a total score range from 0 to 27. Interpretation: 0–4 none–minimal depression; 5–9 mild/subthreshold depression; 10–14: moderate depression; 15–19: moderate–severe; 20–27: severe depression. Internal consistency: *α* = 0.75. Items: *a* referring to “anhedonia,” *c* “sleep disturbance” and *i* “suicidal thoughts,” where also used as individual items
Anxiety	Generalized Anxiety Disorder Questionnaire (GAD–7) [34, 35]	The scale is based on DSM–IV criteria for generalized anxiety disorder, containing seven items on a Likert scale from 0 (*not at all*) to 3 (*nearly every day*) with a total score range from 0 to 21. Interpretation: 0–4: none–minimal anxiety. 5–9: mild anxiety. 10–14: moderate anxiety. 15–21: severe anxiety. Internal consistency: *α* = 0.79
Somatization	Patient Health Questionnaire somatization module (PHQ–15) [36, 37]	The scale is performed by 15 items each one measured by a Likert scale from 0 (*not bothered*) to 2 (*bothered a lot*). The total score ranges from 0 to 30. Interpretation: 0–4 none–minimal somatization; 5–9 mild/subthreshold somatization; 10–14: moderate somatization; 15–30: severe somatization. Internal consistency: *α* = 0.68
Panic disorder	Patient Health Questionnaire panic disorder module (PHQ–PD) [38, 39]	15–item dichotomic (*yes/no*) scale used to determine the presence or absence of panic disorder employing the DSM algorithm. Presence: the first item must be “*yes” and at least one of the next 3 items plus* 4 of the somatic symptoms
Worry	Penn State Worry Questionnaire – Abbreviated (PSQW–A) [40, 41]	8–item–based questionnaire to measure worry, with a maximum score of 40. Each item is a Likert scale from 1 (*it is not typical in me*) to 5 (*it is very typical in me*). Internal consistency: *α* = 0.89
Ruminative response	Ruminative Response Scale – Brooding Subscale (RRS–B) [42, 43]	This scale was used to assess the ruminative thoughts corresponding to the brooding domain. It is a 5–item subscale with a Likert–type response scale from 1 (*almost never*) to 4 (*almost always*). Internal consistency: *α* = 0.76
Metacognition	Metacognitive Questionnaire 30 –Negative Beliefs subscale (MCQ–NB) [44, 45]	The MCQ–NB 5–item subscale of MCQ–30 developed to assess the negative beliefs about uncontrollability and danger, ranging from 5 to 24 measured by Likert scale 1 (*totally disagree*) to 4 (*totally agree*). Internal consistency: *α* = 0.80
Emotional regulation	Emotion Regulation Questionnaire (ERQ) [46, 47]	It is a 10–item scale to assess by two subscales adaptive (ERQ–R, cognitive reappraisal) and maladaptive (ERQ–S, expressive suppression) emotion regulation strategies. Responses are given by a Likert scale from 1 (*strongly disagree*) to 7 (*strongly agree*). Internal consistency: *α* = 0.75
Attentional and cognitive biases	Inventory of Cognitive Activity in Anxiety Disorders Panic–Brief (IACTA–PB) [48]	It is a 5–item scale to measure attentional and cognitive biases by a Likert scale from 0 (*almost never*) to 4 (*almost always*) with maximum punctuation of 20. Internal consistency: *α* = 0.86
Quality of life	World Health Organization Quality of life Instrument–Abbreviated (WHOQOL–Bref) [49, 50]	It is a 26–item scale to assess the quality of life in four domains: physical, psychological, and environmental health and social relationships. The total score ranges from 26 to 130, and scoring is by a Likert scale which ranges from 1 (*very bad*) to 5 (*very good*). Internal consistency: *α* = 0.86
Disability	Sheehan Disability Scale (SDS) [51, 52]	Uses a 5–item Likert scale from 0 (*not at all*) to 10 (*extremely*) that assesses the interference of their symptoms in five daily domains (work, social, and family functioning and stress and perceived social support). 1, 4, and 7 are the cut points for mild, moderate, and high disability, respectively. Internal consistency: *α* = 0.71
Demographics	*Ad hoc*	Self–reported gender, age, marital status (with or without partner), educational level (basic studies, ≤ secondary education and high studies, ≤ university degree) and employment situation (employed or unemployed)
Treatment	*Ad hoc*	Self–reported (treatment as usual or treatment as usual + transdiagnostic group cognitive behavioral therapy)
Psychiatric medication	*Ad hoc*	Currently taken or not antidepressants and/or anxiolytics (yes/no)

### Data analysis

#### Trajectory class modeling

Growth mixture modeling (GMM) [53] is a longitudinal structural equation modeling approach that aims to identify distinct subgroups of individuals in a sample that demonstrate similar patterns of response over time, employing pre-treatment, post-treatment, and follow-up timepoints (3, 6, and 12 months) PHQ-9 scores. To analyze the subgroups of latent classes, GMMs were performed modeling up to six classes of trajectories, identifying different slopes and intercepts for each number of latent classes, which usually had better fit to the data than the average trajectory [54]. To determine the optimal number of classes, each model (*k*) was compared to the previous model (*k –* 1) on the following recommended model fit statistics: the Vuong-Lo-Mendell-Rubin likelihood ratio test (VLMR-LRT), where a *p*-value of < 0.05 indicates the *k* model is a better fit for the data than the *k*−1 model, the Akaike information criterion (AIC) and the Bayesian information criterion (BIC), for which the lowest value between models indicates better fit. Finally, the entropy value of each model was considered, where scores range from 0 to 1 to indicate the accuracy of classification into latent classes, with a value ≥ 0.8 indicating that at least 80% of the individuals were correctly classified into latent classes. Scores between 0.8 and 0.4 indicate medium accuracy and ≤ 0.4 low accuracy [55].

GMM analysis was conducted in Mplus version 8.7 [30]. Missing PHQ-9 data were handled using full information maximum likelihood and the expectation maximization algorithm in Mplus [56].

#### Association of patient and treatment characteristics with trajectory class

Once the optimum class solution was observed, patients were allocated to the trajectory that they had the highest likelihood of membership before associations between patient characteristics and trajectory was assessed. In accordance with Rothman [57], the multinomial logistic regression analyses were used without making adjustments for multiple comparisons in order to facilitate the exploration of potential associations between baseline patient characteristics (sociodemographic, clinical, cognitive-emotional, disability and quality of life, and the influence of the treatment received) with each of the identified trajectory classes (see Table 1 for list of variables). SPSS version 27 was used for these analyses [58].

## Results

### Descriptive statistics

A total sample of 483 patients of the original RCT [9] met the inclusion criteria for this analysis. Of those, 483 had completed pre-treatment and post-treatment assessments, 414 at 3 months, 361 at 6 months, and 316 at 12 months. Table 2 presents the characteristics of the sample.

**Table 2.** Descriptive statistics of patients baseline characteristics

	Total (*N* = 483)	Recovery (*n* = 310)	Late recovery (*n* = 49)	Chronic (*n* = 58)	Relapse (*n* = 66)	*p*-value (chi square or ANOVA)
	Mean (*SD*)	Mean (*SD*)	Mean (*SD*)	Mean (*SD*)	Mean (*SD*)	
Age	44.69 (11.25)	44.32 (11.08)	44.35 (11.38)	47.38 (10.89)	44.35 (12.18)	.289
PHQ–9	14.13 (4.96)	13.57 (4.51)	16.49 (4.73)	18.47 (3.72)	15.92 (4.58)	<.001
PHQ–15	14.43 (4.62)	13.48 (4.45)	14.92 (4.57)	17.76 (4.17)	15.64 (4.18)	<.001
GAD–7	12.67 (4.41)	11.69 (4.32)	13.96 (3.93)	15.34 (3.68)	13.97 (4.3)	<.001
SDS	23.87 (9.29)	22.36 (9.14)	28.22 (9.14)	26.47 (8.82)	25.48 (8.84)	<.001
WHOQOOL–BREF	2.88 (.79)	3.03 (.79)	2.76 (.72)	2.34 (.55)	2.76 (.84)	<.001
PSWQ–A	29.99 (6.68)	28.87 (6.5)	31.08 (6.17)	33.09 (6.16)	31.74 (7.07)	<.001
RRS brooding	13.36 (3.56)	12.7 (3.36)	14.88 (3.21)	14.93 (3.88)	13.92 (3.6)	<.001
IACTA Brief	8.37 (5.18)	7.9 (5.08)	8.51 (5.57)	9.44 (5.35)	9.53 (5.02)	.039
ERQ suppression	15. 51 (5.9)	14.83 (5.76)	16.38 (5.36)	17.84 (6.14)	15.95 (6.14)	.002
ERQ reinterpretation	25.35 (6.87)	25.52 (6.99)	24.93 (6.35)	26.34 (6.22)	24 (7.26)	.25
MCQ negative beliefs	16.26 (4.01)	15.82 (3.91)	16.61 (3.98)	16.82 (4.15)	17.54 (4.08)	.007
Anhedonia	1.78 (.92)	1.64 (.93)	1.92 (.84)	2.24 (.80)	1.92 (.88)	<.001
Sleep disturbance	1.9 (.99)	1.75 (.98)	2.1 (.92)	2.28 (.93)	2.12 (.95)	<.001
	*n* (%)	*n* (%)	*n* (%)	*n* (%)	*n* (%)	
Gender						.549
Female	393 (81.4)	247 (79.3)	43 (87.8)	48 (82.8)	55 (83.3)	
Male	90 (18.6)	63 (27.7)	6 (12.2)	10 (17.2)	11 (16.7)	
Marital status						.1
With partner	339 (70.2)	224 (72.3)	33 (67.3)	33 (56.9)	49 (74.2)	
Without partner	144 (29.8)	86 (27.7)	16 (32.7)	25 (43.1)	17 (25.8)	
Educational level						.039
Basic studies	345 (71.4)	208 (67.1)	38 (77.6)	45 (77.6)	54 (81.8)	
High studies	138 (28.6)	102 (32.9)	11 (22.4)	13 (22.4)	12 (18.2)	
Employment status						.562
Employed	255 (52.8)	160 (51.6)	29 (59.2)	28 (48.3)	38 (57.6)	
Unemployed	228 (47.2)	150 (48.4)	20 (40.8)	30 (51.7)	28 (42.4)	
Treatment group						<.001
TAU	231 (47.8)	121 (39)	33 (67.3)	44 (75.9)	33 (50)	
TAU + TDG–CBT	252 (52.2)	189 (61)	16 (32.7)	14 (24.1)	33 (50)	
Antidepressant use						<.001
No	364 (75.4)	255 (82.3)	32 (65.3)	33 (56.9)	44 (66.7)	
Yes	119 (24.6)	55 (17.7)	17 (34.7)	25 (43.1)	22 (33.3)	
Anxiolytic use						.252
No	302 (62.5)	203 (65.5)	27 (55.1)	36 (62.1)	36 (54.6)	
Yes	181 (37.5)	107 (34.5)	22 (44.9)	22 (37.9)	30 (45.5)	
PHQ–PD						.027
Absence	351 (72.7)	235 (75.8)	34 (69.4)	33 (56.9)	49 (74.2)	
Presence	132 (27.3)	75 (24.2)	15 (30.6)	25 (43.1)	17 (25.8)	
Suicidal thoughts						<.001
Absence	291 (60.2)	216 (69.7)	19 (38.8)	21 (36.2)	35 (53)	
Presence	192 (39.8)	94 (30.3)	30 (61.2)	37 (63.8)	31 (47)	

Abbreviations: ERQ, Emotional Regulation Questionnaire; GAD-7, Generalized Anxiety Disorder-7; IACTA, Inventory of Cognitive Activity in Anxiety Disorders; MCQ, Metacognition Questionnaire; PHQ-9, Patient Health Questionnaire-9; PHQ-15, Patient Health Questionnaire-15; PSWQ, Penn State Worry Questionnaire; RRS, Rumination Response Scale; SD, standard deviation; SDS, Sheehan Disability Scale; TAU, treatment as usual; TDG-CBT, transdiagnostic group cognitive-behavioral therapy; WHOQOL, World Health Organization Quality of Life.

### Trajectories of depressive symptoms

**Figure 1.**
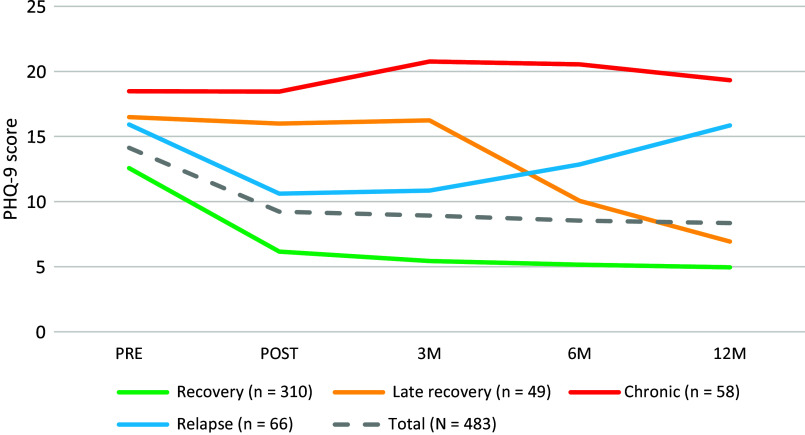
Depression trajectories.

A four-class model was selected as the optimum solution according to the BIC criteria (Table 3). However, according to the VLMR-LRT *p*-value criterion, the best fit to the data was the two-class model because it is the only one with significant differences from the previous class. Nevertheless, according to Nylund et al. [59], in case of discrepancies between criteria, the BIC should be chosen given that it is the most consistent indicator. Class allocation resulted in the following trajectory groups (Figure 1).

**Table 3.** Results of growth mixture modeling analysis

Class solution	Log-likelihood	H0scaling	AIC	BIC	Adj-BIC	VLMR-LRT *p*-value	Entropy	Classification (*n* per profile)
1	−6206.574	1.2586	12441.148	12499.668	12455.233	-	-	483
2	−6177.310	1.4168	12380.621	12434.961	12393.700	0.0006	0.805	98/385
3	−6161.775	1.3240	12357.550	12428.610	12374.653	0.1201	0.811	373/17/93
**4**	**−6138.489**	**1.4207**	**12318.978**	**12406.758**	**12340.106**	**0.2430**	**0.745**	**310/49/58/66**
5	−6129.509	1.2841	12309.018	12413.519	12334.171	0.2045	0.691	268/44/72/37/62
6	−6115.632	1.1880	12289.264	12410.485	12318.441	0.0570	0.731	36/148/30/42/192/35

Abbreviations: Adj-BIC, sample size-adjusted Bayesian information criterion; AIC, Akaike information criterion; BIC, Bayesian information criterion; VLMR-LRT, Vuong-Lo–Mendell–Rubin likelihood ratio test.

Notes: Model fit indices for the favored model are in bold.

*Class 1-recovery* (*n* = 310; 64.18%): It is characterized by moderate symptoms at baseline, (PHQ-9mean = 12.6; *SD* = 4.5), a pronounced decrease at post-treatment (PHQ-9mean = 6.1; *SD* = 4), and a gradual continuation of symptom reduction at follow-up: 3 months (PHQ-9mean = 5.4; *SD* = 3.2), 6 months (PHQ-9mean = 5.2; *SD* = 3.6), and 12 months (PHQ-9mean = 4.9; *SD* = 3.4).

*Class 2-late recovery* (*n* = 49; 10.15%): It is characterized by moderate–severe symptoms at baseline (PHQ-9mean = 16.5; *SD* = 4.7), post-treatment (PHQ-9mean = 16; *SD* = 4.9), and 3-month follow-up assessments (PHQ-9mean = 16.2; *SD* = 4.1), although it shows a gradual symptom reduction was observed at 6 (PHQ-9mean = 10.1; *SD* = 4.5) and 12-month follow-up assessments (PHQ-9mean = 6.9; *SD* = 3.5).

*Class 3-chronic* (*n* = 58; 12.0%): It is characterized by moderate–severe symptoms at baseline (PHQ-9mean = 18.5; *SD* = 3.7) and maintaining similar levels throughout all assessments: post-treatment (PHQ-9mean = 18.4; *SD* = 5.1), 3 months (PHQ-9mean = 20.8; *SD* = 3.6), 6 months (PHQ-9mean = 20.5; *SD* = 3.5), and 12 months (PHQ-9mean = 19.3; *SD* = 4.4).

*Class 4-relapse* (*n* = 66; 13.7%): It is characterized by moderate–severe depressive symptoms at baseline (PHQ-9mean = 15.9; *SD* = 4.6), a decrease at post-treatment (PHQ-9mean = 10.6; *SD* = 4.2), near to the cut-off point (PHQ-9mean = 10), and a gradual increase of symptoms during the follow-up assessments: 3 months (PHQ-9mean = 10.8; *SD* = 3.3), 6 months (PHQ-9mean = 12.8; *SD* = 3.4), and 12 months (PHQ-9mean = 15.8; *SD* = 3.4).

### Associations of baseline variables with trajectory class

The associations between baseline characteristics and depression trajectories were analyzed using multinomial regression models. The Recovery class was used as the reference group as it was the most common trajectory (Table 4).

**Table 4.** Associations between baseline characteristics and PHQ-9 trajectory classes 2, 3, and 4 relatives to class 1 (recovery)

Baseline predictor	Late recovery OR (95%CI) & *p*-value	Chronicity OR (95%CI) & *p*-value	Relapse OR (95%CI) & *p*-value
Age	1.004 (.969–1.04), *p* = .829	1.049 (1.006–1.094), *p* = .026*	.989 (.960–1.019), *p* = .474
Gender			
Female	2.213 (.773–6.336), *p* = .139	.642 (.224–1.839), *p* = .409	1.228 (.539–2.797), *p* = .625
Male	Ref.	Ref.	Ref.
Marital status			
With partner	1.049 (.488–2.257), *p* = .902	.532 (.233–1.213), *p* = .133	1.268 (.626–2.567), *p* = .510
Without partner	Ref.	Ref.	Ref.
Educational level			
Basic studies	1.903 (.831–4.359), *p* = .128	1.257 (.509–3.104), *p* = .620	2.281 (1.091–4.766), *p* = .028*
High studies	Ref.	Ref.	Ref.
Employment status			
Employed	1.901 (.935–3.864), *p* = .076	1.181 (.540–2.583), *p* = .676	1.557 (.853–2.839), *p* = .149
Unemployed	Ref.	Ref.	Ref.
GAD–7	1.119 (1.01–1.24), *p* = .030*	1.204 (1.070–1.354), *p* = .002*	1.061 (.973–1.157), *p* = .181
PHQ–15	1.013 (.928–1.104), *p* = .778	1.217 (1.101–1.346), *p* < .001*	1.083 (1.005–1.166), *p* = .036
PHQ–PD			
Absence	.899 (.383–2.108), *p* = .806	.389 (.157–.962), *p* = .041*	1.620 (.765–3.429), *p* = .208
Presence	Ref.	Ref.	Ref.
Anhedonia	.793 (.511–1.230), *p* = .300	1.245 (.757–2.046), *p* = .388	.937 (.653–1.344), *p* = .724
Sleeping disturbances	1.33 (.906–1.952), *p* = .145	1.242 (.798–1.933), *p* = .288	1.382 (.996–1.917), *p* = .053
Suicidal thoughts			
Absence	.394 (.185–.841), *p* = .016*	.441 (.189–1.030), *p* = .059	.724 (.384–1.363), *p* = .317
Presence	Ref.	Ref.	Ref.
SDS	1.074 (1.026–1.124), *p* = .002*	1.005 (.958–1.054), *p* = .840	1.011 (.974–1.049), *p* = .577
WHOQOOL	.786 (.473–1.306), *p* = .353	.296 (.157–.557), *p* < .001**	.723 (.476–1.098), *p* = .128
PSWQ	.993 (.932–1.058), *p* = .830	1.050 (.975–1.131), *p* = .196	1.023 (.968–1.081), *p* = .426
IACTA	.943 (.871–1.021), *p* = .147	.942 (.862–1.030), *p* = .192	1.004 (.939–1.073), *p* = .914
ERQ suppression	1.059 (.996–1.126), *p* = .069	1.117 (1.042–1.197), *p* = .002*	1.042 (.986–1.100), *p* = .143
ERQ reinterpretation	1.003 (.951–1.058), *p* = .914	1.039 (.978–1.103), *p* = .220	.966 (.923–1.011), *p* = .136
MCQ negative beliefs	.917 (.822–1.023), *p* = .120	.897 (.798–1.009), *p* = .070	1.052 (.957–1.156), *p* = .292
RRS	1.178 (1.039–1.335), *p* = .011*	1.134 (.987–1.304), *p* = .076	.977 (.880–1.085), *p* = .663
Treatment group			
TAU	5.178 (2.460–10.902), *p* < .001**	15.424 (6.079–39.136), *p* < .001**	2.029 (1.113–3.698), *p* = .021*
TAU + TDG–CBT	Ref.	Ref.	Ref.
Antidepressant use			
No	.466 (.209–1.035), *p* = .061	.393 (.167–.928), *p* = .033*	.509 (.251–1.033), *p* = .061
Yes	Ref.	Ref.	Ref.
Anxiolytic use			
No	.822 (.391–1.728), *p* = .606	1.985 (.860–4.586), *p* = .108	.784 (.417–1.475), *p* = .450
Yes	Ref.	Ref.	Ref.

Abbreviations: ERQ, Emotional Regulation Questionnaire; GAD-7, Generalized Anxiety Disorder-7; IACTA, Inventory of Cognitive Activity in Anxiety Disorders; MCQ, Metacognition Questionnaire; PHQ-9, Patient Health Questionnaire-9; PHQ-15, Patient Health Questionnaire-15; PSWQ, Penn State Worry Questionnaire; RRS, Rumination Response Scale; SD, standard deviation; SDS, Sheehan Disability Scale; TAU, Treatment as usual; TDG-CBT, Transdiagnostic group cognitive-behavioral therapy; WHOQOL, World Health Organization Quality of Life.

Note: * *p* < 0.05. ** *p* < 0.01.

The likelihood of being in Class 2-late recovery, relative to Class 1-recovery was higher in patients who had: received TAU alone (OR(95%CI) = 5.18(2.46–10.9)); higher baseline scores for depression (OR(95%CI) = 1.20(1.12–1.29)), generalized anxiety (OR(95%CI) = 1.12(1.01–1.24)), ruminative response (OR(95%CI) = 1.18(1.04–1.33)), and disability (OR(95%CI) = 1.07(1.03–1.12)). Besides, absence of suicidal thoughts (OR(95%CI) = .39(.18–.84)) was associated with a lower likelihood of following the late recovery trajectory respective to recovery.

The likelihood of being in trajectory Class 3-chronicity, compared to Class 1-recovery was higher in those patients that had: received TAU alone (OR(95%CI) = 15.42(6.08–39.14)); higher baseline scores for depression (OR(95%CI) = 1.35(1.24–1.46)), somatization (OR(95%CI) = 1.22(1.10–1.35)); generalized anxiety (OR(95%CI) = 1.20(1.07–1.35)), and emotional suppression (OR(95%CI) = 1.12(1.04–1.2)); and those patients with higher age (OR(95%CI) = 1.05(1.01–1.09)). Besides, higher scores on quality of life (OR(95%CI) = .30(.16–.56)), absence of panic disorder symptoms (OR(95%CI) = .39(.16–.96)) and not taking ADM (OR(95%CI) = .39(.17–.93)) were associated with a lower likelihood of following chronicity than recovery.

The likelihood of being in trajectory Class 4-relapse, compared to Class 1-recovery, was higher in patients with had: basic education level (OR(95%CI) = 2.28(1.09–4.77)); received TAU alone (OR(95%CI) = 2.03(1.11–3.7)); and higher baseline scores for depression (OR(95%CI) = 1.17(1.10–1.24)) and somatization (OR(95%CI) = 1.08(1.01–1.17)).

A binomial regression model was constructed to compare Class 2-late recovery and Class 4-relapse due to similar baseline severity and completely different trajectories across time using Class 4-relapse as the reference group. Higher likelihood of being in Class 2-late recovery was observed for those that had received TAU alone (OR(95%CI) = 2.55(1.1–5.93)) higher ruminative response scores (OR(95%CI) = 1.21(1.04–1.39)); and higher disability scores (OR(95%CI) = 1.06(1.01–1.12)). In addition, higher metacognitive response scores (OR(95%CI) = .87(.77–.99)) were associated with a lower likelihood of following late recovery than relapse (see Table 5).

**Table 5.** Associations between baseline characteristics and PHQ-9 trajectory classes 2 – late recovery relative to class 4 – relapse

Baseline predictor	Late recovery OR (95%CI) and *p*-value
Age	1.015 (.975–1.057), *p* = .477
Gender	
Female	1.803 (.538–6.041), *p* = .339
Male	Ref.
Marital status	
With partner	.828 (.335–2.045), *p* = .682
Without partner	Ref.
Educational level	
Basic studies	.853 (.308–2.259), *p* = .722
High studies	Ref.
Employment status	
Employed	1.221 (.539–2.766), *p* = .632
Unemployed	Ref.
GAD–7	1.055 (.938–1.186), *p* = .375
PHQ–15	.935 (.847–1.033), *p* = .186
PHQ–PD	
Absence	.555 (.205–1.499), *p* = .246
Presence	Ref.
Anhedonia	.846 (.510–1.404), *p* = .518
Sleeping disturbances	.963 (.618–1.501), *p* = .866
Suicidal thoughts	
Absence	.545 (.228–1.304), *p* = .173
Presence	Ref.
SDS	1.063 (1.008–1.120), *p* = .023*
WHOQOOL	1.087 (.606–1.948), *p* = .780
PSWQ	.971 (.901–1.047), *p* = .441
IACTA	.940 (.858–1.029), *p* = .177
ERQ suppression	1.017 (.946–1.092), *p* = .652
ERQ reinterpretation	1.038 (.977–1.103), *p* = .229
MCQ negative beliefs	.873 (.767–.990), *p* = .034*
RRS brooding	1.206 (1.044–1.392), *p* = .011*
Treatment group	
TAU	2.552 (1.098–5.933), *p* = .030*
TAU + TDG–CBT	Ref.
Antidepressant use	
No	.914 (.371–2.252), *p* = .845
Yes	Ref.
Anxiolytic use	
No	1.049 (.448–2.458), *p* = .912
Yes	Ref.

Abbreviations: ERQ, Emotional Regulation Questionnaire; GAD-7, Generalized Anxiety Disorder-7; IACTA, Inventory of Cognitive Activity in Anxiety Disorders; MCQ, Metacognition Questionnaire; PHQ-9, Patient Health Questionnaire-9; PHQ-15, Patient Health Questionnaire-15; PSWQ, Penn State Worry Questionnaire; RRS, Rumination Response Scale; SD, standard deviation; SDS, Sheehan Disability Scale; TAU, Treatment as usual; TDG-CBT, Transdiagnostic group cognitive-behavioral therapy; WHOQOL, World Health Organization Quality of Life.

Note: * *p* < 0.05. ** *p* < 0.01.

## Discussion

### Main findings

This study identified four different trajectories of change in depressive symptoms during the 1-year follow-up, and baseline variables associated with each trajectory in patients with mild to moderate depressive symptoms seen in primary care. The overall trajectory across the sample was observed to be moderate symptoms at baseline, followed by a pronounced reduction of symptoms at post-treatment, followed by a gradual reduction during follow-up assessments. The four identified trajectories were named “recovery,” “late recovery,” “relapse,” and “chronicity.” The “recovery” trajectory was the most common across patients (64%), and there was a similar proportion following each of the remaining trajectories (10–12%). The four subgroups of patients differed in several baseline characteristics.

The four trajectories found here were similar to those identified in prior studies in primary care [15, 19], but differ in prevalence, with fewer patients belonging to the chronicity and relapse trajectories here than in prior studies. This could be due to the different amounts of follow-up time in each study, as more relapses tend to occur in studies with longer follow-up times [16]. Additionally, it may be related to the characteristics of the patients. Our study only included patients with mild to moderate depressive symptoms, whereas in other studies, patients with more severe symptoms were also included, and it is well evidenced that severity is associated with chronicity and relapse [11, 23]. Population-based studies have also found heterogeneity in depression symptoms trajectories and an association between baseline severity and chronicity [20–22].

It is noticeable that TAU was associated with an increased likelihood of following chronicity (OR(95%CI) = 15.42(6.08–39.14)), late recovery (OR(95%CI) = 5.18(2.46–10.9)) and relapse (OR(95%CI) = 2.03(1.11–3.7)) than recovery. Therefore, these results could suggest that the addition of TDG-CBT is one of the most important factors to achieve a sustained recovery over time. This is consistent with findings from meta-analyses of primary care studies that have indicated the effectiveness of psychological therapy [10] and better outcomes from psychological therapy compared to TAU [14]. It is also noteworthy that only 26% of the study sample took ADM, 43% of whom followed the chronicity trajectory. Previous studies showed that patients with prescribed ADM had poorer response to psychological treatment even after adjusting for baseline severity [12, 23, 60].

Lower levels of comorbid generalized anxiety symptoms, panic disorder symptoms, and somatization were also associated with a greater likelihood of following the recovery trajectory class. This could be because of the common factors of emotional disorders [61], although both anxiety symptoms and panic disorder have been found to be independently associated with depression treatment outcomes in primary care, even after accounting for depressive symptom severity [12, 13, 23, 29].

Maladaptive emotional regulation strategies are linked to the development and maintenance of emotional disorders [62], so one of the goals of transdiagnostic psychological therapies is helping patients to develop adaptive emotional regulation strategies [61]. Previous studies of the PsicAP trial focused on the mediating effect of cognitive processes and emotional regulation strategies on treatment outcomes had shown that patients assigned to TAU+TDG-CBT had significant changes in emotional regulation strategies (worry, rumination, metacognitive beliefs, and emotional suppression) in post-treatment and in depressive symptoms compared to patients who had received TAU alone [63]. Similar results were also found for rumination in the UK primary care settings finding that a higher pre-treatment level of rumination was associated with a worse prognosis [27]. In the present study, we found that higher baseline levels of emotional suppression were associated with the chronicity trajectory class and higher levels of rumination were associated with the late recovery trajectory.

Participants with a basic education were more likely to follow the relapse trajectory compared to the recovery trajectory, consistent with prior studies [11, 16]. However, this association may be influenced by confounding factors such as lower socioeconomic status, housing and other sociodemographic variables associated with depression treatment prognosis in primary care [26], none of which was possible to adjust for in this study. Other studies in primary care have found an association between being unemployed and chronicity [12, 25]. However, in the present study, unemployment was not associated with any trajectory, which could be due to cultural or social reasons.

Differences between late recovery and relapse trajectories are noteworthy given that both groups started with similar baseline severity, but the trajectories are completely different. Patients who followed the late recovery trajectory were characterized by higher baseline scores in disability and rumination, and lower scores in metacognitive beliefs. They were also more likely to have been allocated to receive TAU alone. The comparison between trajectories such as these has not been carried out in previous studies, and the assessment of cognitive-emotional variables, which is an aspect in which both trajectories differ at baseline, are not usually employed either. It was expected that the relapse group would show higher rumination levels at baseline, like other studies [64]. However, our findings here agree with those that had found that a higher rumination is related with a maintenance and residual symptomatology of depression [62] which could explain that higher rumination levels are related with the trajectory of late recovery.

### Strengths and limitations

This is the first study to investigate trajectories of change in depressive symptoms and associations between pre-treatment patient characteristics and likely trajectory class membership over treatment and up to 1-year follow-up in Spanish primary care services. Likewise, this study is one of the first investigations worldwide based on an RCT that considers longitudinal repeated measures and uses GMM models to identify subgroups with similar patterns in patient trajectories. In addition, this was the first study of psychological therapy to have assessed putative cognitive-emotional mechanisms associated with the onset and maintenance of depression and its resolution during therapy and to consider their associations with different trajectories.

However, there were several limitations to the study. First, a large proportion of patients dropped out following the end of treatment and so did not complete all follow-up assessments, which may have biased the results. It is noteworthy, though, that the rate of drop-out during the post-treatment and follow-up were similar in both treatment groups [9], and similar to the rate reported in other RCTs conducted in primary care according to the meta-analysis by Bortolotti et al. [65]. Second, the study lacks information about the clinical history of the patients; therefore, it is likely that the depressive symptoms did not start at the beginning of the trial, and patients with a chronic course may have been more likely to take antidepressants prior to the trial starting. Moreover, the sample for this study consisted of middle-aged women. It was observed that the prevalence of females with depression in the sample was higher compared to the general population in Spain [66] or in primary care settings [67]. This difference could be attributed to the fact that this profile could be more inclined to participate in an RCT involving group therapy for emotional disorders. Alternatively, GPs may be more inclined to refer women than men to a study of this nature. Consequently, it is possible that this sample may not be fully representative of patients in primary care.

## Conclusions

The present study contributes to a growing and promising area of knowledge on the study of the prognosis of depression and associated baseline characteristics in primary care settings.

These findings highlight the importance of adding TDG-CBT to TAU to improve the chances of recovering from depressive symptoms. Furthermore, it provides a useful tool for the personalization of depression treatment and the identification of subgroups at higher risk based on baseline characteristics. For example, patients exhibiting higher comorbidity, baseline severity of depression, or maladaptive emotional regulation skills were more likely to have a worse prognosis compared to patients with lower scores in these domains. This could have significant implications for the management of depression in primary care and the capacity of these public healthcare services to reduce the burden of depression. While this study offers an initial exploration of personalized treatments, further research, including replication studies and analyses of trajectories per treatment and related characteristics, is necessary before these findings can significantly influence care decisions.

## Supporting information

Prieto-Vila et al. supplementary material Prieto-Vila et al. supplementary material

## Supplementary material

The supplementary material for this article can be found at http://doi.org/10.1192/j.eurpsy.2024.27.
