# Supplementary material for: Long-term depressive symptom trajectories and related baseline characteristics in primary care patients: Analysis of the PsicAP clinical trial
Source: Eur Psychiatry. 2024 Mar 27;67(1):e32. doi: 10.1192/j.eurpsy.2024.27 (PMC11059253; doi:10.1192/j.eurpsy.2024.27)
Supplement: Prieto-Vila et al. supplementary material [file S0924933824000270sup001.docx]

**Supplementary materials**

**Supplementary Table 1.** *Comparison between analytical sample and original sample.*

|  | Total  (N=1061) | Total  (N = 483) | *p-value* (*Student’s t*) |
| --- | --- | --- | --- |
|  | Mean (*SD*) | Mean (*SD*) |  |
| Age | 42.95 (11.82) | 44.69 (11.25) | .009* |
| PHQ-9 | 13.62 (5.37) | 14.13 (4.96) | .068 |
| PHQ-15 | 14.16 (4.84) | 14.43 (4.62) | .267 |
| GAD-7 | 12.29 (4.65) | 12.67 (4.41) | .102 |
| SDS | 23.31 (9.73) | 23.87 (9.29) | .285 |
| WHOQOOL-BREF | 2.91 (.83) | 2.88 (.79) | .553 |
| PSWQ-A | 30.09 (6.77) | 29.99 (6.68) | .774 |
| RRS brooding | 13.56 (3.57) | 13.36 (3.56) | .322 |
| IACTA Brief | 8.15 (5.41) | 8.37 (5.18) | .423 |
| ERQ suppression | 15.47 (6.03) | 15. 51 (5.9) | .924 |
| ERQ reinterpretation | 25.58 (7.62) | 25.35 (6.87) | .597 |
| MCQ negative beliefs | 16.36 (4.37) | 16.26 (4.01) | .640 |
| Anhedonia | 1.7 (.95) | 1.78 (.92) | .142 |
| Sleep disturbance | 1.85 (1.03) | 1.9 (.99) | .348 |
|  |  |  |  |
|  | n (%) | n (%) | *p-value (χ2*) |
| Gender |  |  | .944 |
| Female | 861 (81.1) | 393 (81.4) |  |
| Male | 200 (18.9) | 90 (18.6) |  |
| Marital status |  |  | .013* |
| With partner | 675 (63.6) | 339 (70.2) |  |
| Without partner | 386 (36.4) | 144 (29.8) |  |
| Educational level |  |  | .581 |
| Basic studies | 773 (72.9) | 345 (71.4) |  |
| High studies | 288 (27.1) | 138 (28.6) |  |
| Employment status |  |  | .700 |
| Employed | 572 (53.9) | 255 (52.8) |  |
| Unemployed | 489 (46.1) | 228 (47.2) |  |
| Treatment group |  |  | .380 |
| TAU | 534 (50.3) | 231 (47.8) |  |
| TAU + TDG-CBT | 527 (49.7) | 252 (52.2) |  |
| Antidepressant use |  |  | .659 |
| No | 788 (74.3) | 364 (75.4) |  |
| Yes | 273 (25.7) | 119 (24.6) |  |
| Anxiolytic use |  |  | .693 |
| No | 652 (61.5) | 302 (62.5) |  |
| Yes | 409 (38.5) | 181 (37.5) |  |
| PHQ-PD |  |  | .449 |
| Absence | 766 (72.2) | 351 (72.7) |  |
| Presence | 295 (27.8) | 132 (27.3) |  |
| Suicidal thoughts |  |  | .535 |
| Absence | 658 (62) | 291 (60.2) |  |
| Presence | 403 (38) | 192 (39.8) |  |

*Abbreviations*: *SD* = Standard Deviation; χ2 = Chi Square; *PHQ-9* = Patient Health Questionnaire-9; *PHQ-15* = Patient Health Questionnaire-15; *GAD-7* = Generalized Anxiety Disorder-7; *WHOQOL* = World Health Organization Quality of Life; *SDS* = Sheehan Disability Scale; *PSWQ* = Penn State Worry Questionnaire; *RRS* = Rumination Response Scale; *IACTA* = Inventory of Cognitive Activity in Anxiety Disorders; *ERQ* = Emotional Regulation Questionnaire; *MCQ* = Metacognition Questionnaire; *TAU* = Treatment as usual; *TDG-CBT* = Transdiagnostic group cognitive-behavioral therapy.

**Supplementary Figure 1.** *Flow-chart of the participants of the original and current study.*
